# Development of an E-learning System for the Endoscopic Diagnosis of Early Gastric Cancer: An International Multicenter Randomized Controlled Trial

**DOI:** 10.1016/j.ebiom.2016.05.016

**Published:** 2016-05-17

**Authors:** K. Yao, N. Uedo, M. Muto, H. Ishikawa, H.J. Cardona, E.C. Castro Filho, R. Pittayanon, C. Olano, F. Yao, A. Parra-Blanco, S.H. Ho, A.G. Avendano, A. Piscoya, E. Fedorov, A.P. Bialek, A. Mitrakov, L. Caro, C. Gonen, S. Dolwani, A. Farca, L.F. Cuaresma, J.J. Bonilla, W. Kasetsermwiriya, K. Ragunath, S.E. Kim, M. Marini, H. Li, D.G. Cimmino, M.M. Piskorz, F. Iacopini, J.B. So, K. Yamazaki, G.H. Kim, T.L. Ang, D.M. Milhomem-Cardoso, C.A. Waldbaum, W.A. Piedra Carvajal, C.M. Hayward, R. Singh, R. Banerjee, G.K. Anagnostopoulos, Y. Takahashi

**Affiliations:** a Fukuoka University Chikushi Hospital, Chikushino, Japan; b Osaka Medical Center for Cancer and Cardiovascular Diseases, Osaka, Japan; c Kyoto University, Kyoto, Japan; d Kyoto Prefectural University of Medicine, Kyoto, Japan; e Simon Bolivar Hospital, Bogota, Colombia; f Rio de Janeiro State University, Rio de Janeiro, Brazil; g King Chulalongkorn Memorial Hospital, The Thai Red Cross and Chulalongkorn University, Bangkok, Thailand; h Universidad de la República, Montevideo, Uruguay; i Peking Union Medical College Hospital, Beijing, China; j School of Medicine, Pontificia Universidad Catolica De Chile, Santiago, Chile; k University of Malaya, Kuala Lumpur, Malaysia; l Hospital Rafael Angel Calderon Guardia, CCSS, San Jose, Costa Rica; m Universidad Peruana de Ciencias Aplicadas, Lima, Peru; n Russia National Medical University, Moscow University Hospital, N31, Moscow, Russian Federation; o Pomeranian Medical University, Szczecin, Poland; p Nizhniy Novgorod Cancer Hospital, Nizhniy Novgorod, Russian Federation; q GEDyt Gastroenterologia diagnostica y tratamiento Inst afiliafa a la UBA Buenos Aires, Argentina; r Haydarpasa Numune Training and Research Hospital, Istanbul, Turkey; s Division of Population Medicine, School of Medicine, Cardiff University, Cardiff, United Kingdom; t The American British Cowdray Medical Center. Mexico City, Mexico; u Hospital Nacional Adolfo Guevara Velasco, Cusco, Peru; v i-gastro/Hospital Central de la Fuerza Aerea del Peru, Lima, Peru; w Faculty of Medicine Vajira Hospital, Navamindradhiraj University, Bangkok, Thailand; x NIHR Nottingham Digestive Disease Biomedical Research Unit, Queens Medical Centre, Nottingham University Hospital, Nottingham, United Kingdom; y Kosin University College of Medicine, Busan, Republic of Korea; z Gastroenterology and Operative Endoscopy Unit, Siena University Hospital, Siena, Italy; aa Sichuan Provincial People's Hospital, Sichuan, Academy of Medical Sciences, Chengdu, China; ab Hospital Aleman, Buenos Aires, Argentina; ac Hospital de Clinicas Jose de San Martin, Buenos Aires, Argentina; ad Ospedale S. Giuseppe, ASL Roma 6, Albano L, Rome, Italy; ae National University of Singapore, Singapore, Singapore; af University of Sao Paulo, Sao Paulo, Brazil; ag Pusan National University School of Medicine, Busan, Republic of Korea; ah Changi General Hospital, Singapore, Singapore; ai General Hospital of Goiania, Goiania, Brazil; aj Hospital de Clinicas Jose de San Martin, Buenos Aires, Argentina; ak Hospital Mexico, San Jose, Costa Rica; al Derriford Hospital, Plymouth, United Kingdom; am Lyell McEwin Hospital & University of Adelaide, Adelaide, Australia; an Asian Institute of Gastroenterology, Hyderabad, India; ao Mitera General Hospital, Athens, Greece; ap FAST Inc., Tokyo, Japan

**Keywords:** Endoscopic diagnosis, Gastric cancer, E-learning, International multicenter randomized controlled trial

## Abstract

**Background:**

In many countries, gastric cancer is not diagnosed until an advanced stage. An Internet-based e-learning system to improve the ability of endoscopists to diagnose gastric cancer at an early stage was developed and was evaluated for its effectiveness.

**Methods:**

The study was designed as a randomized controlled trial. After receiving a pre-test, participants were randomly allocated to either an e-learning or non-e-learning group. Only those in the e-learning group gained access to the e-learning system. Two months after the pre-test, both groups received a post-test. The primary endpoint was the difference between the two groups regarding the rate of improvement of their test results.

**Findings:**

515 endoscopists from 35 countries were assessed for eligibility, and 332 were enrolled in the study, with 166 allocated to each group. Of these, 151 participants in the e-learning group and 144 in the non-e-learning group were included in the analysis. The mean improvement rate (standard deviation) in the e-learning and non-e-learning groups was 1·24 (0·26) and 1·00 (0·16), respectively (*P* < 0·001).

**Interpretation:**

This global study clearly demonstrated the efficacy of an e-learning system to expand knowledge and provide invaluable experience regarding the endoscopic detection of early gastric cancer (R000012039).

## Highlights

• This report establishes that an e-learning system on the Internet can improve the diagnostic ability of endoscopists.
• Countless endoscopists worldwide can access the system to learn how to make an endoscopic diagnosis of early gastric cancer.
• The e-learning system could be modified to provide education regarding endoscopic diagnosis in other organs.

This is the first report to demonstrate how an e-learning system based on the Internet can improve the diagnostic ability of gastrointestinal endoscopists worldwide. There is no limit to the number of endoscopists who can access the system and benefit from this opportunity to learn how to make an endoscopic diagnosis of early gastric cancer. This e-learning system could be modified to provide education regarding endoscopic diagnosis in other organs such as the large intestine and the esophagus, as well as the stomach. It may contribute to human welfare and health by reducing the mortality from gastrointestinal cancer.

## 1 Introduction

Almost one million new cases of gastric cancer were estimated to have occurred throughout the world in 2012 (952,000 cases, 6.8% of total new cancer cases), making it the fifth most common malignancy after cancers of the lung, breast, colorectum and prostate (GLOBOCAN, 2012). Gastric cancer is the third leading cause of death from cancer among both sexes worldwide (723,000 deaths, 8.8% of total cancer deaths). Most patients with gastric cancer are diagnosed at an advanced stage, with an overall 5-year survival rate of approximately 28% (American Cancer Society. Cancer facts and figures, 2014). Early detection is the key to improving the survival of gastric cancer patients (Leung et al., 2008). Upper gastrointestinal endoscopy is a widely accepted procedure for early detection of gastric cancer. However, in many countries, endoscopists have limited opportunities to acquire the techniques, knowledge and experience which are imperative for the endoscopic detection of early gastric cancer (EGC) when only subtle mucosal morphology is apparent (Veitch et al., 2015). In contrast, endoscopists in Japan have more such opportunities thereby enabling them to detect subtle lesions that suggest EGC.

In order to overcome these problems, we have developed an Internet-based e-learning system which is in English, and which is available anywhere in the world, and at any time of the day, so that clinicians worldwide can learn how to detect EGC (Yao, 2013). Recently, advanced imaging endoscopy techniques have become a topic for discussion in various academic meetings or publications (American Gastroenterological Association (AGA), 2008). Nevertheless, white-light endoscopy is still the most common practice throughout the world. This e-learning system is therefore dedicated to teaching diagnosis using white-light conventional endoscopy alone (Yao, 2012).

We hypothesized that if endoscopists could acquire the detailed “knowledge, techniques and experience” essential for the early detection of gastric cancer through this e-learning system, then the detection rate of early-stage gastric cancer would increase throughout the world (Veitch et al., 2015). Accordingly, we investigated the feasibility of this e-learning system to improve the ability of endoscopic detection of EGC among endoscopists outside Japan.

## 2 Materials and Methods

### 2.1 Study Design

Global e-Endo Study Team (GEST) was organized to develop an e-learning system for improving the detection rate of EGC among endoscopists worldwide. This study was conducted as an international, randomized, controlled trial to evaluate the effectiveness of the e-learning system. The study was conducted in line with the Consolidated Standards of Reporting Trials (CONSORT) statement (Schulz et al., 2010) and the Declaration of Helsinki (World Medical Association Declaration of Helsinki, 2013).

The study protocol was approved by the institutional review board of Fukuoka University Chikushi Hospital, Japan (R12-060,dated February 6, 2013), and was registered as Clinical Trial No. UMIN R000012039. Written informed consent was obtained from all participating endoscopists.

### 2.2 Participants

We recruited endoscopists from 35 countries around the world between March 2013 and November 2013. Inclusion criteria were 1) ability of the web browser on a participant's computer to display and to operate sample contents of the e-test and e-learning system; 2) sufficient English skills to understand the materials of the e-test and e-learning system; 3) provision of a fully completed pre-study questionnaire sheet; and 4) provision of a signed consent form for participation in this study. Medical practitioners who did not complete the pre-test or whose pre-test score was 80% or more were excluded from the study because the e-learning system was aimed at providing training for those who had not previously received adequate training in the endoscopic diagnosis of EGC. A specific username and password were assigned to each participant to enable e-test results to be collected via the Internet and to control access to the e-learning system during the e-learning period.

## 3 Interventions

### 3.1 Pre-test Evaluation

**Fig. 1:**
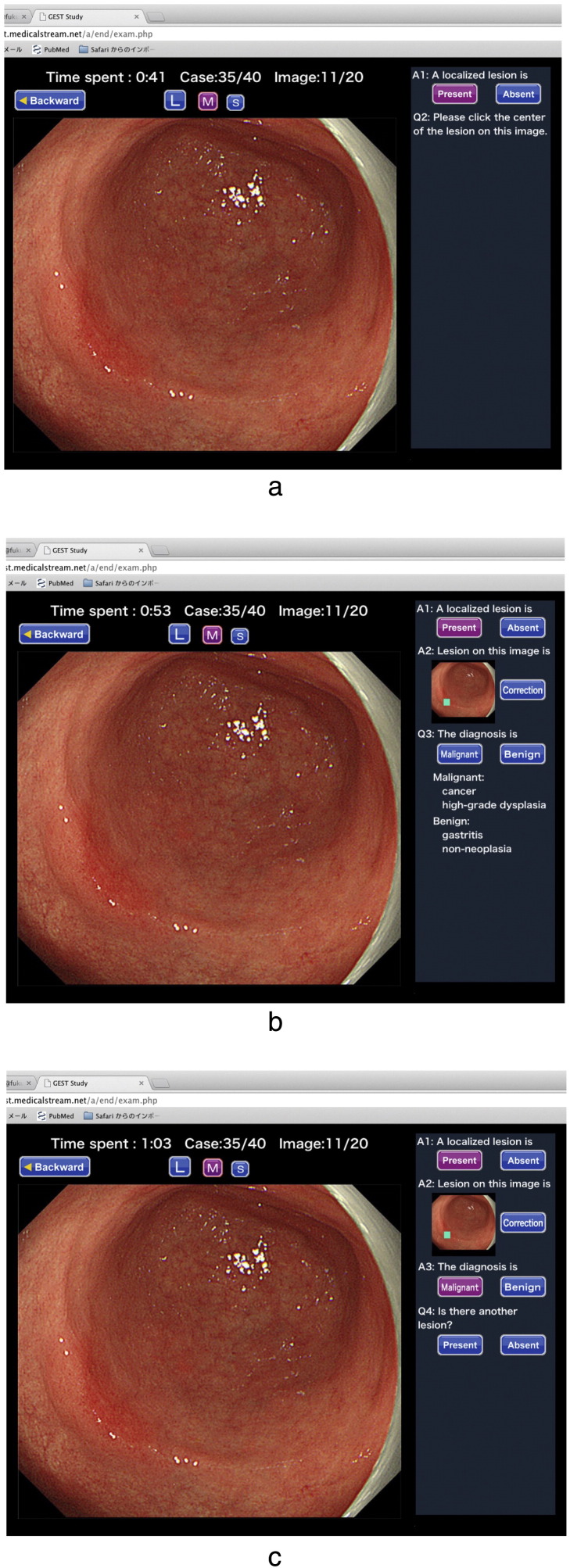
An example of the pre- and post-test. a. When the participant starts the test, an endoscopic image appears. The first question is whether or not a localized lesion is present. If the participant clicks “present” as shown by “A1” in the slide, the second instruction is for the participant to click on the center of the detected lesion on the image as shown by “Q1”. b. The third question is whether the detected lesion is malignant or benign. c. The participant is then offered the chance to identify any additional lesion.

Participating endoscopists undertook a pre-test via the Internet between February 1st 2014 and February 28th 2014. The participants viewed 40 sets of endoscopic images on their web browser. Each set of endoscopic images contained 18 to 24 images that had been systematically taken during screening endoscopy to record the whole gastric mucosa in a single patient, according to systematic screening protocol for the stomach (Veitch et al., 2015, Yao, 2013). All endoscopy images were acquired using a high-definition electronic endoscopy system (EVIS Lucera Spectrum System, Olympus Co. Ltd., Tokyo, Japan) and high-definition upper gastrointestinal endoscopes (GIF-H260; GIF-H290, Olympus Co. Ltd.). The 40 patients consisted of 20 patients with EGC and 20 patients with non-cancerous findings. Each lesion was histopathologically confirmed as either cancer or non-cancer. The histopathologic diagnosis was based on the revised Vienna classification (Schlemper et al., 2000); C1 (negative for neoplasia), C2 (indefinite for neoplasia) and C3 (mucosal low-grade neoplasia) were diagnosed as non-cancer, whereas C4 (mucosal high-grade neoplasia) and C5 (submucosal invasion of neoplasia) were diagnosed as cancer (Ezoe et al., 2011). The EGC was defined as cancer whose depth of invasion is limited to the submucosal layer (Japanese Gastric Cancer Association, 2011). The lesions are present in some, but not all, of the images. The images have sufficient quality to permit the differentiation of cancer from non-cancer (Fig. 1). As shown in Fig. 1, the participants were asked to analyze each image in each set for:

(1). whether a lesion was present or not in the image;
(2). if present, the location of the lesion; and
(3). endoscopic diagnosis (cancer or non-cancer).

Test scores were marked for correct answers to (1), (2) and (3), up to a total of 100 points. To avoid potential bias, participants were not informed about the number of cancerous cases among the test sets.

### 3.2 Allocation

Eligible participating endoscopists were randomly allocated into two groups - an e-learning group and a non-e-learning group - based on stratification of pre-test scores, experience of endoscopy (number of procedures performed), whether the endoscopist was an endoscopy nurse or medical doctor, medical institution and country. The block randomization method was used for randomization using Excel software (Japanese version, Microsoft Co. Ltd., Tokyo). The randomized allocation was performed by the statistician (H.I.) at the data center who was not blinded for allocation. Because the statistician has never been acquainted with the participants' information and performed randomization automatically based on the above-mentioned stratification rule, we did not think that this would result in any bias for randomized allocation. The participants who were allocated to the e-learning group were allowed access to the e-learning system via the Internet from May 1st, 2014 until June 15th, 2014 (e-learning period), whereas those in the non-e-learning group were prevented from accessing the e-learning system.

### 3.3 E-learning System

**Fig. 2:**
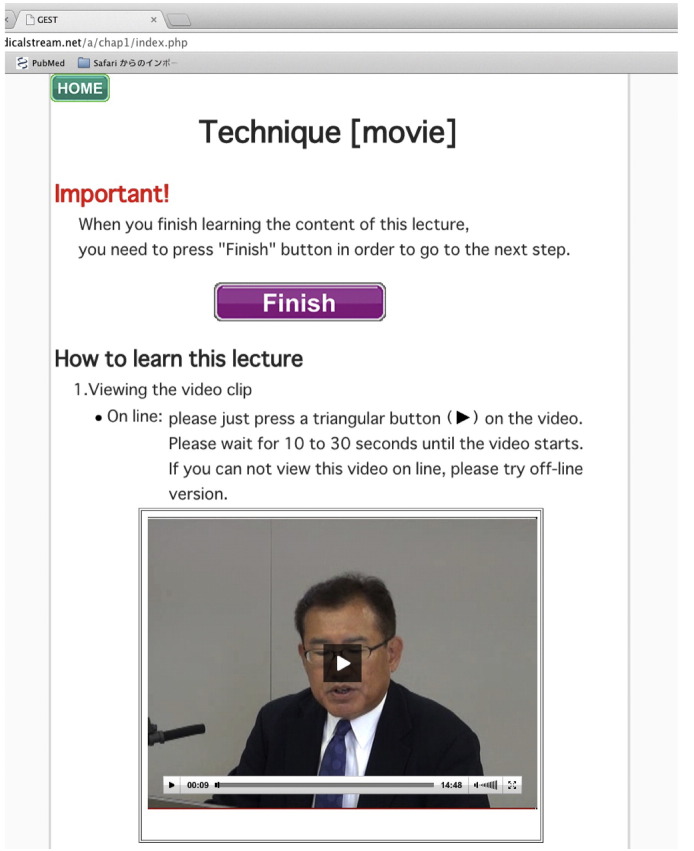
An example of a video clip. On the lecture page, lecture video clips can be viewed online or can be downloaded.

**Fig. 3:**
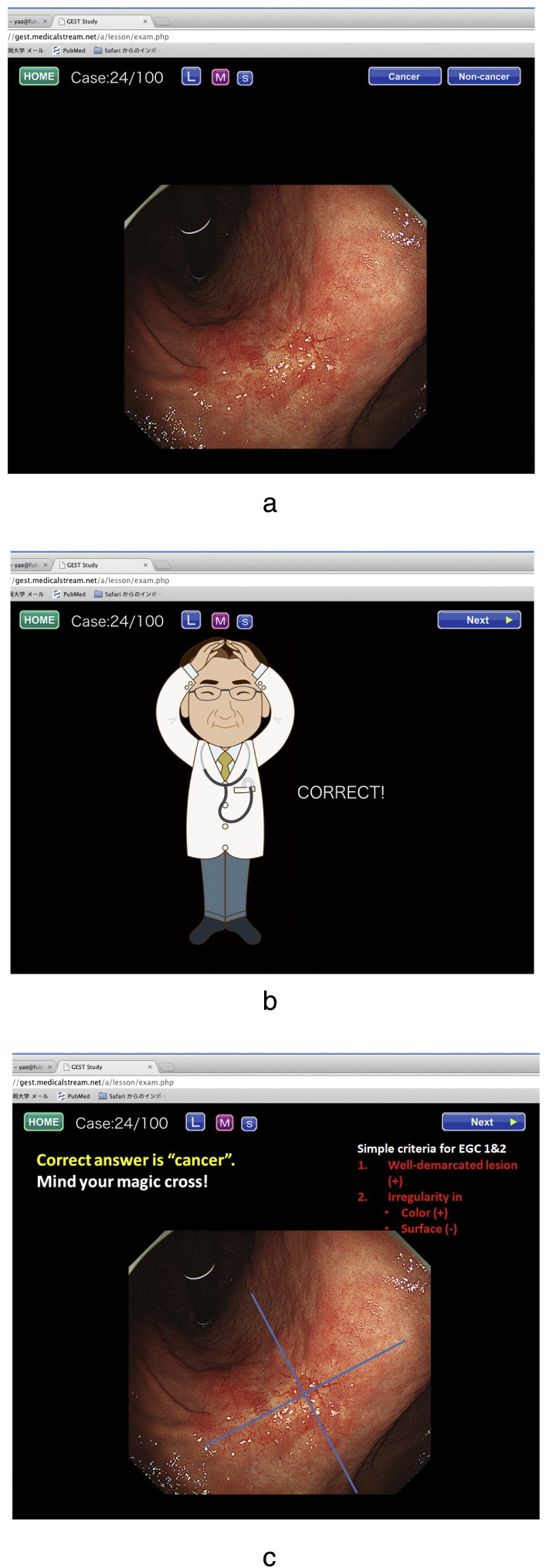
An example of the self-exercise tests for diagnosis of 100 cases. a. One case comprises a set of three slides. The 1st slide showes one endoscopic photo where one lesion is present. First,the participant should click to choose whether the lesion is cancer or non-cancer. b. Immediately after clicking on their choice, an illustration indicating whether the answer is correct or incorrect appears as the 2nd slide. c. The 3rd slide indicates brief instructions on how to characterize the endoscopic findings so as to make a correct diagnosis, and shows the original endoscopic image again.

The e-learning system was composed of video lectures about basic techniques and knowledge, and self-exercise tests to accumulate experience (Fig. 2, Fig. 3). The content was originally constructed for the purpose of improving the ability of a participant to endoscopically detect EGC by conventional white light endoscopy. The e-learning content comprised:

1. Technique
  – Lecture (video clips & slides) (Fig. 2)
2. Knowledge
  – Test (ten questions without answers)
  – Lecture (video clips & slides) (Fig. 2)
  – Test (ten questions with answers and descriptions)
3. Experience: 100 cases for EGC detection training (Fig. 3)
  – Mock test (ten cases with scores and no answers)
  – Random version of the 100 cases
  – Systematic version of the 100 cases
  – Random version of the 100 cases
  – Mock test (ten cases with scores and answers)

In the lecture regarding techniques, we included the following subjects: (1) absolute necessity to complete ideal preparation with mucolytic and anti-foaming agents, (2) recommendation to use antispasmodic agent such as hyoscine butylbromide to inhibit peristalsis and to improve mucosal visualization and (3) importance of avoiding blind spots (Veitch et al., 2015, Yao, 2013). In order to avoid blind areas during observation, we demonstrated a standardized procedure. This includes adequate air insufflation to extend the gastric lumen in order to separate folds, the rinsing of mucus and bubbles from the mucosal surface, and minimally required standard practice for screening procedures to enable mapping observation of the whole gastric mucosa. That procedure was originally proposed as a systematic screening protocol for the stomach (SSS), as described previously (Veitch et al., 2015, Yao, 2013).

In the lecture regarding knowledge, we demonstrated the following subjects: (1) endoscopic appearance of normal gastric mucosa vs. that of abnormal high-risk condition for gastric cancer, such as atrophic gastritis or intestinal metaplasia, (2) how to detect suspicious lesions in the stomach and (3) how to characterize a detected lesion according to macroscopic type, i.e. gastritis-like (G), ulcerative (U) and polypoid (P) lesions. This diagnostic system was named the GUP system and was originally proposed for use in this e-learning system.

In order to improve the endoscopist's ability to detect subtle mucosal gastric cancer, experience is imperative, in addition to good techniques and knowledge. After learning the techniques and acquiring knowledge, if participants can then accumulate experience with numerous endoscopic images of cases with cancerous lesions and of cases with non-cancerous lesions, they should then be able to make a correct diagnosis at a glance. From such a perspective, we developed a self-exercise test program which includes images of 100 cases for detection. The cases comprise 50 early gastric cancers and 50 non-cancerous lesions. We prepared 100 sets of images, each set comprising 3 images for one case of either cancer or non-cancer, as shown in Fig. 3. The participant should make continuous effort to make a diagnosis of the presented images one after another throughout the 100 cases. The cases are arranged either in random order or systematic order according to the GUP system. Repeated practice of quick question and quick answer in 100 cases offers the participants substantial experience in discerning between cancer and non-cancer in their own minds.

The participants who were allocated to the non-e-learning group were taught nothing during the e-learning period.

### 3.4 Post-test Evaluation

After the e-learning period, all participants (both e-learning and non-e-learning groups) received the post-test between June 16th, 2014 and July 31st, 2014. The post-test evaluation used the same format and methodology as the pre-test evaluation, however the participants did not know how the post-test questions differed from the pre-test questions, in order to minimize any carry-over effect.

The e-tests and the e-learning content were prepared by the lead researcher (K. Y.). Endoscopic images were supplied by the Department of Endoscopy at Fukuoka University Chikushi Hospital. All images were taken during actual clinical practice. All patient information (including patient ID, name, gender, age and date of examination) was removed, and each image was allocated a new number for tracking purposes. The e-test and the e-learning system were both originally constructed by an information technology engineer (Y. T.)

### 3.5 Study Outcomes

The primary outcome was the difference in the degree of improvement in e-test results between the “e-learning” and “non-e-learning” groups. The degree of improvement was determined as post-test result (score)/pre-test result (score). The secondary outcomes were the difference in the degree of improvement in the e-test according to pre-test score, experience of endoscopy and geographical difference. The participants were divided into low and high pre-test groups according to the mean pre-test score of the whole baseline group. Experience of endoscopy was divided into less experienced (< 8 years) and experienced (≥ 8 years) groups based on the median years of experience (7 years). Geographical region was divided into Asia-Pacific, Europe and Latin America.

### 3.6 Sample Size

In this trial, we decided to invite as large a number of participants as possible without calculation of an actual sample size in order to expand the benefit of e-learning. In a usual clinical trial, we need to minimize the number of participants who receive random allocation from an ethical viewpoint. However, in this study, there was no significant disadvantage for the participants because eventually, all participants were able to receive the e-learning.

### 3.7 Statistical Analysis

The data were expressed as mean (standard deviation, SD). The difference in score improvement between the two groups was examined by an independent-sample t-test, with a *P* value of < 0.05 indicating statistical significance. All analyses were performed using SPSS software (IBM SPSS Statistics Ver. 20, Chicago, IL).

## 4 Results

### 4.1 Participant Flow and Baseline Characteristics

**Fig. 4:**
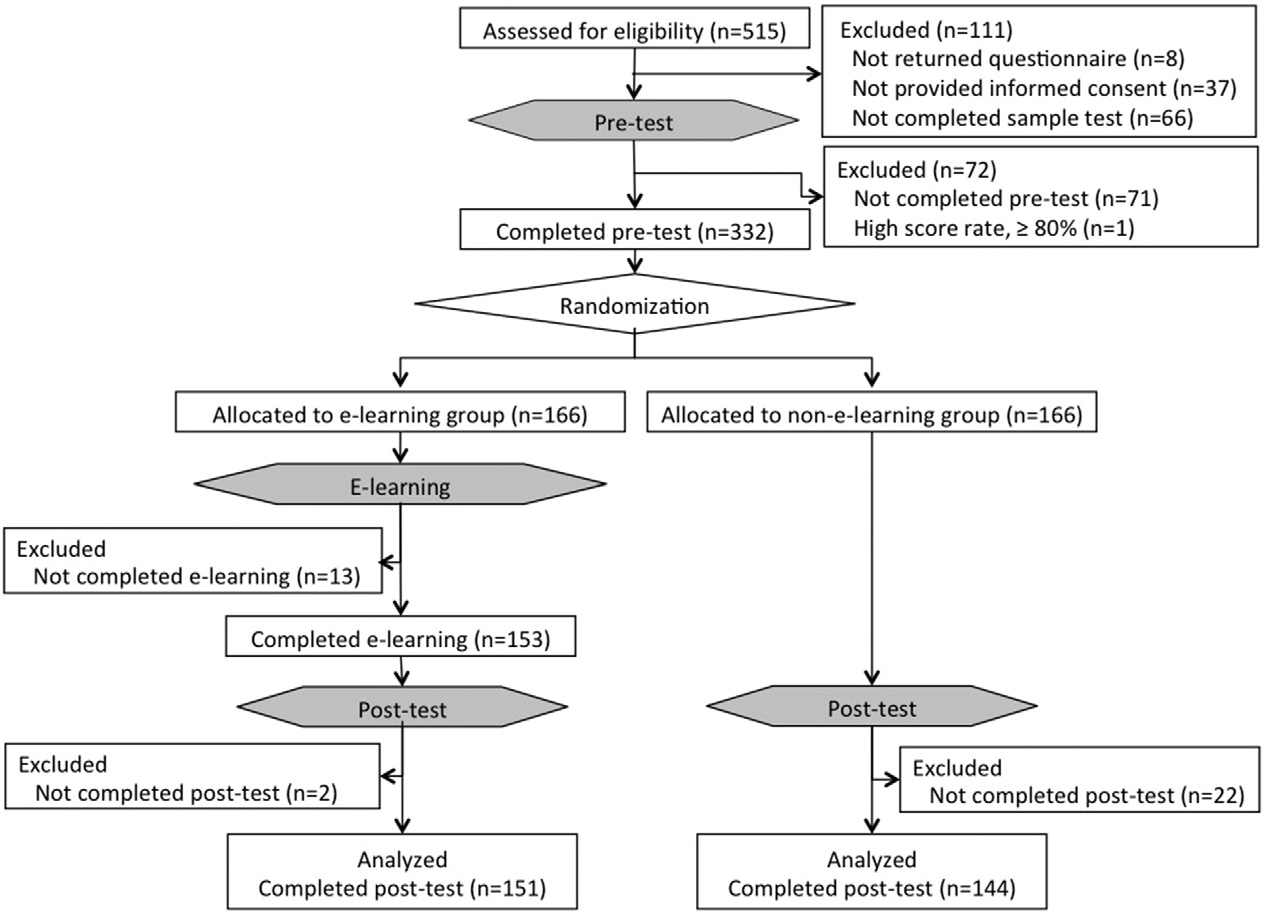
Details regarding enrollment of participants, randomization and e-tests. IC: informed consent.

Among the 515 endoscopists from 35 countries assessed for eligibility, 332 participants from 27 countries who met the inclusion criteria completed the pre-test and were enrolled in the study. Of these participants, 166 were allocated to the e-learning group and 166 to the non-e-learning group. During the e-learning period, 151 participants in the e-learning group completed both e-learning and the post-test, while 144 participants in the non-e-learning group completed only the post-test (Fig. 4). The data of 151 participants in the e-learning and 144 in the non-e-learning groups were analyzed. The baseline characteristics were similar in the e-learning and the non-e-learning groups (Table 1).

### 4.2 Study Outcomes

The mean pre-test score (SD) in the e-learning group was 51·4 (10·9), which improved to 62·2 (11·2) at the post-test. On the other hand, the mean pre-test score in the non-e-learning group was 52·6 (10·3), which remained almost unchanged at 52·4 (11·4) at the post-test. Accordingly, the mean rate of improvement of the test score was significantly better in the e-learning group than in the non-e-learning group [1·24 (0·26) vs. 1·00 (0·16), *P* < 0·001, Table 2].

Subgroup analyses according to the pre-test score, experience of endoscopy and geographical region showed that the mean rate of improvement of the test score in the e-learning group was significantly higher than that in the non-e-learning group among all subgroups (Table 2).

There were no reports of unpleasant effects, such as the creation of too much overload on the living activities of the participants during the trial. In addition, no technical trouble was encountered with the e-test and the e-learning system that caused participation to be discontinued during the trial.

## 5 Discussion

### 5.1 Key Results

This is the first international randomized controlled trial to show that an e-learning system is effective for increasing the ability of endoscopists worldwide to expand their knowledge and gain invaluable experience regarding the endoscopic detection of EGC, as demonstrated by an improvement in their test score. According to the subgroup analyses, the e-learning system was effective irrespective of the pre-test score, the endoscopist's experience or geographical area.

### 5.2 Efficacy of E-learning System

In this study, we constructed an e-learning system based on the Internet. To date, conventional instruction has been conducted on a one-to-one basis by tutorial teaching. Hands-on seminars are also efficient for passing on knowledge and skills hand-to-hand. Nevertheless, the efficacy of such instruction and seminars is limited to small numbers of trainees. Lectures can provide instruction to a larger audience, but the impact is still limited to perhaps a few hundred attendees. Printed literature has been believed to be the most effective tool in the field of mass education. However, in the case of endoscopy, it is difficult to provide content that can effectively teach technique as well as promote knowledge. As constructed in this study, an e-learning system based on the Internet offers a huge advantage over the above-mentioned conventional teaching methods in that there is no limit on the number of learners who can participate. In addition, we were able to upload educational content using an originally developed system which includes an original concept. The outcome in this study clearly shows that good practice based on good knowledge can certainly improve the ability of the participants. It has been reported that a lecture from an expert does improve the ability of an endoscopist to make a correct diagnosis (Mabe et al., 2014). However, to the best of our knowledge, this is the first report to demonstrate that an e-learning system based on the Internet can improve the diagnostic ability of gastrointestinal endoscopists worldwide.

### 5.3 E-learning Content

The e-learning content focused on just three subjects, these being technique, knowledge and experience. The Internet is in fact a useful method for distributing content to an unlimited number of people, but the quality of the content is obviously paramount. Among those three subjects, endoscopists can acquire knowledge and technique by attending conventional lectures or hands-on seminars. However, it is difficult for learners to accumulate experience by a single lecture or hands-on seminar. Therefore, we incorporated 100 cases of EGC detection training into this e-learning system. We believe that simple but repetitive practice is useful for maintaining ability in any learning opportunity. Mabe et al. indicated that the learning effect may decrease if endoscopists do not continue their learning practice (Mabe et al., 2014). An Internet-based e-learning system has the advantage that endoscopists can repeat the practice and maintain their ability wherever and whenever they wish.

### 5.4 Limitations

One of the limitations of this trial is that the primary outcome was not an improvement of the detection rate of EGC in actual clinical practice, but an improvement of test scores. Nevertheless, we have already started a clinical study to investigate the improvement of EGC detection rate in real clinical practice after finalizing this trial as described in the protocol (UMIN: R000012039). In that study, the number of detected EGCs during the post-e-learning period (one-year after this study) will be compared with that during the pre-e-learning period (the one- year period prior to this study). Another limitation is that this Internet-based system was not designed to be interactive. We hope to improve the system thereby enabling it to accept questions or allow discussion from the participants. Finally, this system has only an English version currently, however other major language versions could be provided in the future.

### 5.5 Generalizations

The content of this e-learning system did not utilize advanced imaging endoscopy, but conventional white-light endoscopy alone which can be available in any facility in the world. If we were to upload this system onto an official website and offer participation free of charge, then unlimited numbers of endoscopists worldwide would have the opportunity to learn how to make an endoscopic diagnosis of EGC. Furthermore, this e-learning system could be modified to provide education regarding endoscopic diagnosis in other organs, such as the large intestine and the esophagus, as well as the stomach. It may contribute to human welfare and health by reducing the mortality from gastrointestinal cancer.

In conclusion, as clearly shown by the increased test scores of participants in the e-learning group, this multicenter randomized controlled trial has successfully demonstrated that an Internet-based e-learning system was effective in enabling health practitioners around the world to improve their knowledge and experience with regard to making an endoscopic detection of EGC.

## Funding

The Central Research Institute of Fukuoka University (I) and JSPS Core-to-Core Program (B. Asia-Africa Science Platforms).

## Author Contributions

1. Yao K: Conception and design, analysis and interpretation of data, participant recruitment, and drafting the article
2. Uedo N: Conception and design, analysis and interpretation of data, participant recruitment
3. Muto M: Conception and design
4. Ishikawa H: Conception and design, analysis and interpretation of data
5. Cardona HJ: Participant recruitment, acquisition of data, revising the manuscript and final approval of the article
6. Castro Fiho EC: Participant recruitment, acquisition of data, revising the manuscript and final approval of the article
7. Pittayanon R: Participant recruitment, acquisition of data, revising the manuscript and final approval of the article
8. Olano C: Participant recruitment, acquisition of data, revising the manuscript and final approval of the article
9. Yao F: Participant recruitment, acquisition of data, revising the manuscript and final approval of the article
10. Parra-Blanco A: Participant recruitment, acquisition of data, revising the manuscript and final approval of the article
11. Ho SH: Participant recruitment, acquisition of data, revising the manuscript and final approval of the article
12. Avendano AG: Participant recruitment, acquisition of data, revising the manuscript and final approval of the article
13. Piscoya A: Participant recruitment, acquisition of data, revising the manuscript and final approval of the article
14. Fedorov E: Participant recruitment, acquisition of data, revising the manuscript and final approval of the article
15. Bialek AP: Participant recruitment, acquisition of data, revising the manuscript and final approval of the article
16. Mitrakov A: Participant recruitment, acquisition of data, revising the manuscript and final approval of the article
17. Caro L: Participant recruitment, acquisition of data, revising the manuscript and final approval of the article
18. Gonen C: Participant recruitment, acquisition of data, revising the manuscript and final approval of the article
19. Dolwani S: Participant recruitment, acquisition of data, revising the manuscript and final approval of the article
20. Farca A: Participant recruitment, acquisition of data, revising the manuscript and final approval of the article
21. Cuaresma LF: Participant recruitment, acquisition of data, revising the manuscript and final approval of the article
22. Bonilla JJ: Participant recruitment, acquisition of data, revising the manuscript and final approval of the article
23. Kasetsermwiriya W: Participant recruitment, acquisition of data, revising the manuscript and final approval of the article
24. Ragunath K: Participant recruitment, acquisition of data, revising the manuscript and final approval of the article
25. Kim SE: Participant recruitment, acquisition of data, revising the manuscript and final approval of the article
26. Marini M: Participant recruitment, acquisition of data, revising the manuscript and final approval of the article
27. Li H: Participant recruitment, acquisition of data, revising the manuscript and final approval of the article
28. Cimmino DG: Participant recruitment, acquisition of data, revising the manuscript and final approval of the article
29. Piskorz MM: Participant recruitment, acquisition of data, revising the manuscript and final approval of the article
30. Iacopini F: Participant recruitment, acquisition of data, revising the manuscript and final approval of the article
31. So JB: Participant recruitment, acquisition of data, revising the manuscript and final approval of the article
32. Yamazaki K: Participant recruitment, acquisition of data, revising the manuscript and final approval of the article
33. Kim GH: Participant recruitment, acquisition of data, revising the manuscript and final approval of the article
34. Ang TL: Participant recruitment, acquisition of data, revising the manuscript and final approval of the article
35. Milhomem-Cardoso DM: Participant recruitment, acquisition of data, revising the manuscript and final approval of the article
36. Waldbaum CA: Participant recruitment, acquisition of data, revising the manuscript and final approval of the article
37. Piedra Carvajal WA: Participant recruitment, acquisition of data, revising the manuscript and final approval of the article
38. Hayward CM: Participant recruitment, acquisition of data, revising the manuscript and final approval of the article
39. Singh R: Participant recruitment, acquisition of data, revising the manuscript and final approval of the article
40. Banerjee R: Participant recruitment, acquisition of data, revising the manuscript and final approval of the article
41. Anagnostopoulos GK: Participant recruitment, acquisition of data, revising the manuscript and final approval of the article
42. Takahashi Y: Engineering, acquisition of data, revising the manuscript and final approval of the article

## Figures and Tables

**Table 1:** Characteristics of the participants at baseline.

		E-learning group (n = 166)	Non-e-learning group (n = 166)
Qualification			
	Medical doctor	163	164
	Endoscopy nurse	3	2
Pre-test score (%)			
	Median	51.8	52.2
	(range)	(28.0 - 71.6)	(27.5 - 74.6)
Experience of endoscopy (yr)			
	1-3	52	52
	4-7	38	33
	8-10	30	15
	11-	46	66
Area	Country		
Asia-Oseania			
	Australia	0	1
	China	8	8
	India	1	0
	Korea	3	3
	Malaysia	5	5
	Singapore	3	3
	Thailand	9	8
	Turkey	4	4
Europe			
	England	8	8
	Italy	3	4
	Poland	5	4
	Portugal	2	2
	Russia	8	9
Latin America			
	Argentina	13	12
	Bolivia	5	5
	Brazil	28	28
	Chile	7	7
	Colombia	23	23
	Costa Rica	7	7
	Ecuador	2	1
	El Salvador	1	1
	Guatemala	0	1
	Mexico	3	2
	Paraguay	0	1
	Peru	11	11
	Uruguay	6	6
	Venezuela	1	2

**Table 2:** Degree of improvement in test score between the e-learning group vs. the non-e-learning group.

	E-learning group			Non-e-learning group			
n	Mean rate	SD	n	Mean rate	SD	
Overall	151	1·24	0·26	144	1·00	0·16	a
Lower score group	86	1·34	0·29	80	1·03	0·18	a
Higher score group	65	1·19	0·14	64	1·03	0·11	a
Less experienced group	84	1·28	0·26	72	0·98	0·17	a
More experienced group	67	1·19	0·26	72	1·03	0·14	a
Asia-Oceania	32	1·33	0·34	30	1·05	0·17	a
Europe	22	1·18	0·24	21	0·94	0·23	b
Latin America	97	1·23	0·23	93	1·00	0·13	a

a. *P* < 0.001 for e-learning group vs. no e-learning group.

b. *P* = 0.002 for e-learning group vs. no e-learning group.
